# Human Herpesvirus 6 Associated Encephalitis with Fulminant Brain Edema in a Previously Healthy Child

**DOI:** 10.7759/cureus.8018

**Published:** 2020-05-07

**Authors:** Fabricio Sevilla-Acosta, Juliana Araya-Amador, Adriana Ulate-Campos

**Affiliations:** 1 Pediatrics, Hospital Nacional De Niños "Dr. Carlos Sáenz Herrera", San José, CRI; 2 Pediatrics, Hospital La Anexión, Nicoya, CRI; 3 Pediatric Neurology, Hospital Nacional De Niños "Dr. Carlos Sáenz Herrera", San José, CRI

**Keywords:** acute encephalitis, brain edema, children, human herpes virus 6

## Abstract

Human herpesvirus 6 (HHV-6) is an important cause of roseola and febrile seizures in children. However, it is also a rare cause of encephalitis, most common in immunosuppressed children. We describe a case of HHV-6 encephalitis with fulminant brain edema in a previously healthy child. This severe HHV-6 clinical case with lethal brain edema is the second reported in the literature in a previously healthy child.

## Introduction

Human herpesvirus (HHV)-6 and HHV-7 are ubiquitous T-lymphotropic viruses that infect most humans. Infections with either agent occur primarily during childhood. Seroprevalence of HHV-6 reaches >80% in children aged two years and older. HHV-6 and HHH-7 have been associated with a variety of clinical manifestations including fever, rash, and seizures. Immunocompromised hosts, particularly transplant recipients, are at an increased risk of symptomatic primary or reactivation disease associated with HHV-6 or HHV-7 [1].

The role of HHV-6 and HHV-7 in central nervous system (CNS) diseases is an area of ongoing investigation. The range of CNS manifestations ascribed to these viruses includes asymptomatic infection, febrile seizures, seizure disorders, meningitis, meningoencephalitis, facial palsy, vestibular neuritis, demyelinating disorders, hemiplegia, and, rarely, fatal encephalitis [1].

HHV-6 usually causes roseola in children and follows a benign course. However, severe sequels that affect the CNS can occur, and rarely death [2]. We describe a case of HHV-6 encephalitis with fulminant brain edema in a previously healthy child. To the best of our knowledge, there is only one reported case of a rapidly progressing brain edema in a previously healthy child, making our case uncommon [3]. We hope that in presenting the case, physicians will consider HHV-6 as a pathogen that could have a negative outcome even in immunocompetent children.

## Case presentation

A nine-month-old girl, with unremarkable clinical history, presented to our emergency room with a history of three days of fever and one day of diarrhea. Three hours prior to admission to our center, the patient suffered a generalized tonic-clonic seizure that lasted almost 30 minutes. It required two doses of IV diazepam, one dose of phenytoin, and endotracheal tube protection of the airway. The patient then presented cold extremities and liquid stools. It was necessary to reanimate with 50 cc/kg of normal saline initially and then another 50 cc/kg of crystalloids prior transfer to our center. The patient was admitted to our center in evident hypovolemic shock, with mydriatic pupils, no spontaneous movements despite no sedation, and absence of brainstem reflexes.

Laboratory findings showed metabolic acidosis (pH: 7.18; pCO_^2^_: 26.5 mm Hg; HCO_^3^_: 10 mEq/L; lactate: 2.4 mmol/L), pancytopenia (hemoglobin: 10.8 g/dL; leucocytes: 2,320/mm^3^, neutrophils: 33%; bands: 16%; lymphocytes: 46%, and platelets: 122 x 103/mm^3^), negative C-reactive protein, normal renal function test and electrolytes, elevated hepatic transaminases (AST: 888 U/L; ALT: 232 U/L), and coagulopathy (PT: 39%; ATTP: 73.2 s). 

**Figure 1:**
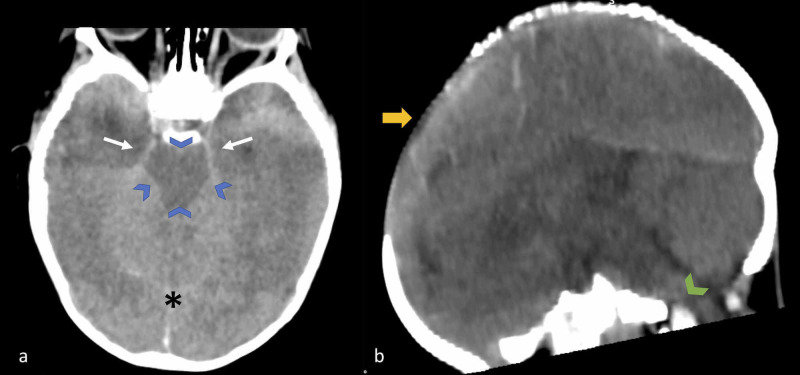
Brain CT scan of a nine-month-old child with fulminant brain edema. (a) Axial view: diffuse brain edema, obliteration of the peri-mesencephalic cisterns (blue arrowheads), temporal lobes edema without herniation (white arrows), and obliteration of superior cerebellum cistern (asterisk). (b) Coronal view: diffuse brain edema with protrusion of anterior fontanelle (yellow arrow) and collapse of pre-pontine cistern (green arrowhead). CT, computed tomography

CNS computed tomography (CT) revealed severe diffuse cerebral edema, collapsed lateral ventricles, thigh basal cisterns and, no clear differentiation between gray and white matter (Figure 1).

Cefotaxime and acyclovir were started empirically. Cerebral spinal fluid (CSF) analysis revealed HHV-6 using BIOFIRE® FilmArray® Meningitis/Encephalitis Panel (bioMérieux, Marcy-l'Étoile, France). Neurologic death was declared five hours later. CSF and bloodstream cultures were negative. Autopsy was carried out and reported herniation of cerebellar tonsils, cerebral edema, and meningoencephalitis due to HHV-6 by polymerase chain reaction.

## Discussion

HHV-6 and HHV-7 are considered uncommon causes of CNS infection and may occasionally cause encephalitis in young infants; however, the clinical syndrome and incidence are not well defined. In immunosuppressed hosts, reactivation is associated with a worse outcome such as encephalitis, hepatitis, or graft rejection. In immunocompetent hosts, this persistent infection is generally of no consequence [4]. However, in the last decade, there have been reports that these viruses are not as benign as we have always thought and that they are able to cause a severe disease even in immunocompetent hosts. 

As testing for HHV-6 in CSF is more readily available using the BIOFIRE® FilmArray® Meningitis/Encephalitis Panel, it is easier to diagnose previously undetermined etiology of encephalitis in children [5]. However, clinical significance of the finding of HHV-6 in CSF should be analyzed in the context of the patient. As an example, in 2012, the FEBSTAT [Consequences of Prolonged Febrile Seizures in Childhood] study was conducted and the authors concluded that HHV-6 and HHV-7 account for one-third of febrile status epilepticus in children aged one month to five years [6]. This suggests that the detection of HHV-6 in CSF is frequent and is not always associated with a clinical diagnosis of encephalitis in children. In our case, the child presented with a clinical diagnosis of encephalitis, and histopathological findings confirmed the clinical diagnosis and HHV-6 was the only pathogen detected in the CSF analysis made. 

Although HHV-6 is a frequently detected pathogen in CSF of children, its role in the development of encephalitis is not clear, and severe cases have been reported recently. In 2020, You studied patients with HHV-6 encephalitis. Of the nine patients positive for HHV-6, three (33.3%) had encephalitis, three (33.3%) had meningitis, one (11.1%) had complex febrile seizures, and two (22.2%) had fever alone. All patients with HHV-6 and encephalitis had neurologic sequelae. Of the three patients with HHV-6 associated encephalitis, two were treated with IV immunoglobulin (IVIg) and antiviral agents and had less severe sequelae. The remaining one patient, who was treated only with antiviral agents, had severe sequelae and developed Lennox-Gastaut syndrome [7]. This study suggests that combinations of antiviral agents and immunomodulatory agents (e.g., steroids, IVIg) during early stages of HHV-6 infection may minimize neurologic sequelae. No mortality was reported in this case series.

To the best of our knowledge, there has been only one case of a patient who died because of HHV-6 encephalitis due to associated brain edema. This case was published in 2018 by Miyahara et al. They reported on a previously healthy fifteen-month-old infant who presented high fever with a generalized seizure twelve hours after onset. There were no apparent neurologic deficits and she was diagnosed with a simple febrile seizure and discharged. The patient persisted with high fever but was reported to be doing well and did not present respiratory symptoms or rash [3]. No gastrointestinal symptoms were described, in contrast to our patient who had a history of diarrhea. She was found dead on the third day of the disease [3]. The postmortem CT scan showed diffuse brain swelling and narrowing of the cerebral sulcus. Brain autopsy showed massive brain edema with brain softening [3]. Unlike our case, no cerebral herniation was found on autopsy. In this case, the subtype HHV-6B was found in both the CSF and in the autopsied brain. Interestingly, the number of HHV-6B was found to be four to five times higher in the hippocampus in relation to other tested brain areas [3]. The number of virus copies in distinct regions of the brain was not studied in our case, but we believe that carrying out such study can lead to further understanding of the pathophysiology of both primary and reactivated HHV-6.

HHV-6 is naturally resistant to acyclovir. Three drugs initially developed to target human cytomegalovirus infection have been shown to be efficient against HHV-6 infection both in vitro and in vivo: ganciclovir, foscarnet, and cidofovir [8]. In our case, we think that what led to our patient’s death was the severe brain edema, refractory to medical treatment. In severe encephalitis, brain edema may lead to a fatal course due to increased intracranial pressure (ICP) not responding to conventional methods (osmotherapy, hyperventilation, barbiturates, and steroids). Decompressive craniectomy has recently been proposed to control intractable elevated ICP for various neurologic diseases including encephalitis.﻿ Singhi et al. recommend that a decompressive craniectomy be carried out in all focal CNS infections as soon as the intracranial hypertension becomes refractory to standard medical treatment or if there is ongoing worsening in the clinical condition as a result [9]. In 2000, Taferner et al. reported four cases of encephalitis with refractory brain edema treated with craniectomy with dural augmentation. They conclude that this treatment approach in cases of severe space-occupying encephalitis, not only saves the patient's life but also leads to favorable long-term outcome. However, no children were included in this study and the youngest patient was 17 years old [10]. Our patient presented a rapidly deteriorating condition and may have benefited from such procedure in initial stages, but when she came to our center, she already had an absence of brain stem reflexes, and neurologic death was declared hours later. Timely recognition of refractory intracranial hypertension and surgical decompression in children with encephalitis could be life-saving.

The exact fatality rate of HHV-6-associated encephalitis at the time of primary infection remains unclear. Disabling sequelae (e.g., visual impairment, speech disturbance, persistent hemiplegia, quadriplegia, and mental retardation) are frequent in children after an HHV-6 encephalitis or meningoencephalitis course [11]. Our case is rare, and we hope that in presenting the case, physicians will consider HHV-6 as an important pathogen that can also have a negative outcome in immunocompetent children. Treatment should be considered early in the disease and prompt treatment of brain edema is essential for the survival of the patient.

## Conclusions

Although HHV-6 is a rare cause of encephalitis in children, when it occurs with brain edema, it can be lethal, even in immunocompetent children. Outcome depends on the aggressive treatment of brain edema. Treatment with antiviral agents and immunomodulatory agents (e.g., steroids, IVIg) during early stages of HHV-6 infection should be considered, and prompt treatment of brain edema is essential for the survival of the patient.
